# A five-year retrospective evaluation of a faculty research fellowship programme at the medical college of Georgia

**DOI:** 10.1007/s40037-016-0303-3

**Published:** 2016-10-13

**Authors:** Tasha R. Wyatt, Kelli Braun, Lance Evans, Alexis Rossi, Paul M. Wallach, Lara M. Stepleman

**Affiliations:** Educational Innovation Institute, Medical College of Georgia at Augusta University, Augusta, USA

**Keywords:** Fellowship, Educational research, Faculty development

## Abstract

**Introduction:**

In institutional assessments of faculty, scholarly activity is often cited as a deficiency. Faculty lack the training and resources needed to produce peer-reviewed, quality scholarship. Although a variety of formats have been suggested and used to fill this void, fellowships are a commonly used format to foster educational leaders within institutions. In 2010, the Educational Innovation Institute at the Medical College of Georgia created an educational research fellowship to address this need.

**Methods:**

To assess the success of our programme, we compared all graduating fellows’ current curriculum vitae (CVs) with the version submitted at the time of their application, looking for educational scholarship produced during and after their participation in the fellowship. Qualitative data sources, such as article reflections, mid-fellowship surveys, and exit surveys were analyzed to identify the mechanisms that contributed to their success. The constant comparative method was used to identify themes and patterns.

**Results:**

A comparison of CVs collected at the time of application with a current CV indicate the 11 participants produced: 60 presentations at regional or national meetings, 16 peer reviewed publications, received funding for 7 grants supporting educational research, and won 7 national research awards. Our qualitative analysis identified three major mechanisms: 1) dedicated time to conducting educational research, 2) opportunities to engage with others, and 3 ) understanding the differences between educational and clinical research.

**Discussion:**

Previous criticisms of fellowships include faculty not producing educational scholarship after completing their programme. Our retrospective analysis indicates our research fellowship was successful in developing physicians and clinical educators to become educational researchers. What was most useful was having dedicated time to work with others interested in producing educational scholarship, and expert guidance in understanding the differences between clinical and educational research. The most challenging aspect of conducting education research was their need to use conceptual frameworks and learning theory in their work. Implications for this study include the need for a strong curricular focus on the differences between clinical and educational research for any fellowship programme.

## What this paper adds

- In institutional assessments of faculty, scholarly activity is often cited as a deficiency. Fellowship programs are often created to fill this gap. In 2010, the Medical College of Georgia created an educational research fellowship with the explicit goal of nurturing faculty members’ development as educational researchers. In an internal evaluation of the fellowship, our 5‑year retrospective analysis demonstrated that the programme assisted 11 fellows to produce nearly 90 pieces of scholarship. Our retrospective analysis identifies ways the fellowship leveraged faculty success and the challenges fellows experienced with moving from clinical bench scientists to educational researchers.

## Introduction

In 2011, a national survey was conducted of medical education faculty fellowships in medical schools and hospitals across the US [1]. The results of this study indicate there is an increasing interest in utilizing medical educational fellowships as a means for training educational leaders. This has been prompted in part by the Accreditation Council for Graduate Medical Education and the Liaison Committee on Medical Education, which require medical faculty to engage in scholarly activity for accreditation and promotion. In institutional assessments of faculty, scholarly activity is often cited as a deficiency in a large part because faculty lack the training and resources needed to produce peer-reviewed, quality scholarship [2].

Although a variety of formats have been suggested and used to fill this void, such as workshops and seminars [3], faculty development fellowships are a commonly used format to foster educational leaders within institutions [4, 5]. Fellowships are defined as single cohorts of medical teaching (and sometimes other health professions) faculty who participate in extended activities to better design, implement, and disseminate research, create innovative curriculum, increase teaching skills [6], in addition to other skill sets to conduct research [7]. It is estimated that the majority of American medical schools have some form of faculty fellowship programme, although they differ significantly in scope and focus.

The purpose of this paper is to evaluate the effectiveness of the educational research fellowship offered by the Educational Innovation Institute at the Medical College of Georgia (MCG), which has demonstrated itself to be a supportive vehicle for developing educational scholarly activity in medical and health professions school faculty. In this paper, we report evaluation data collected as part of a retrospective analysis on what was successful, alongside some of the mechanisms that seemed to have contributed to its success. We also discuss some of the challenges our fellows had in learning to become educational researchers and our revisions for evaluation as we move forward.

In the first section, we provide a short history of the programme and how it has developed over the last five years followed by a retrospective analysis using both quantitative and qualitative data. Finally, we propose programmatic revisions and future directions for research and development as we continue to improve MCG’s fellowship.

## A short history of MCG’s faculty development fellowship programme

In 2008, the Dean of the School of Medicine at MCG gathered a team of educators and educational researchers to develop an educational discovery institute that paralleled other translational science discovery institutes forming within the institution. The Educational Discovery Institute, whose name was later changed to the Educational Innovation Institute, was developed with the vision of becoming a national leader in advancing health education research, innovation and discovery and scholarship. The four aims of the Educational Innovation Institute were to 1) develop educational discovery that enhances learning and improves patient outcomes; 2) apply rigorous methodology to the study of educational research questions; 3) use a team approach to foster university-wide collaboration; and 4) build an external network of collaborators and funding portfolios [8].

In 2010, the Educational Innovation Institute’s first educational research fellowship track was developed with the explicit goal of nurturing faculty members’ career progression by providing training in health sciences education research and fostering career development. The mission of this fellowship track is to facilitate participants’ development as 1) producers of health professions education research, 2) advocates for educational research within their own departments and the broader Augusta University community, and 3) coaches, mentors, and leaders to other faculty interested in or engaged in health professions educational research. These aims are in line with many other medical schools which have similar programmes [9].

Initially, the fellowship was designed for MCG’s faculty as a one-year programme, in which fellows met weekly for two hours. The Educational Innovation Institute paid for 10 % full-time equivalents and participants’ home departments added an additional 10 % towards a full day of fellowship training and research. The overall curriculum focused on the basics of educational research and eventual development of a project suitable for peer review and presentation/publication upon completion of the programme. The specific sessions focused on understanding the basic principles of educational research with topics that included: defining research and scholarship in educational research, navigating the Institutional Review Board, collecting and analyzing data, and the process of development and refinement of a research question. The curriculum also included an overview of various study designs and how to choose different designs based on the research question. It also covered psychometric issues such as measurement, reliability, and validity. In 2011, the fellowship was expanded to schools outside MCG to include all health sciences educator faculty with the intent of creating a rich inter-professional educational research experience with increased opportunities for cross-fertilization between participants.

Further development occurred in 2014 when the fellowship was expanded from one year to two years and meeting arrangements shifted from weekly to monthly for fellows in their second year. Given participants’ time constraints, the shift from one to two years was designed to allow participants further time to develop. In total 12 fellows were admitted into the programme, however one dropped out midway through the fellowship due to time constraints. Now, five years into the research fellowship, the Educational Innovation Institute has conducted a retrospective analysis on the extent to which the fellowship has met the needs of both the fellows’ and the institution’s desire to create educational researchers.

## Methods

To assess the effect of the Educational Innovation Institute fellowship on fellows’ ability to produce scholarship, we analyzed quantitative data on participants’ productivity during and after the fellowship and qualitative data to understand what contributed to fellows’ productivity. We began by contacting the 11 fellows to request a current copy of their curriculum vitae. This version was then compared with the one submitted at the time of application and then analyzed for educationally focused peer-reviewed scholarship. These data helped us to see what was produced before entry into the programme and compare it to what was produced during and after graduation. In addition, we analyzed qualitative data collected from several data sources including participants’ reflections, surveys, and semi-structured exit interviews to better understand what may have contributed to the fellows’ success. Constant comparative method [10] was used to code and analyze the data; this a method is recommended as an analytical lens when trying to make sense of new data, such as the mechanisms that may have contributed to the fellows’ success in this programme. Institutional Review Board approval was given both at the time of initial data collection and re-approved at the time of this analysis.

## Results

A comparison of the curriculum vitae indicates the 11 participants produced: 60 presentations at regional or national meetings, 16 peer-reviewed publications, received funding for 7 grants supporting educational research, and won 7 national research awards (Table 1). This comparative analysis indicated that all participants initially came to the fellowship programme without having produced any educational scholarship and therefore 100 % of their total educational scholarly activity was produced during or after participants completed the fellowship programme. This initial analysis indicates that the fellowship provided the kinds of support fellows needed. In an effort to better understand what contributed to the fellows’ success, our analysis identified three major mechanisms. Two are structural in nature and one is attributed to the curriculum itself. The three themes are: 1) dedicated time to conducting educational research, 2) opportunities to engage with others, and 3) understanding the differences between educational and clinical research. The next section includes representative comments found in our analysis.

**Table 1:** Type of scholarship by fellow, year and department

Fellow	Year	Department	Presentations	Publications	Grants	Awards
1	2010	Paediatrics	6	1	3	1
2	2010	Paediatrics	7	0	2	1
3	2011	Cell biology	13	0	0	0
4	2011	Psychiatry	9	8	2	3
5	2011	Paediatrics	9	1	0	0
6	2012	Internal medicine	2	0	0	0
7	2012	Physical therapy	2	4	0	1
8	2012	Family medicine	2	1	0	0
9	2013	Ob/Gyn	7	0	0	1
10	2014	Internal medicine	0	1	0	0
11	2014	Cell biology	3	0	0	0

## Dedicated time to conduct research

The fellows highlighted the importance of dedicated time to work on educational research as one reason for their success. Having dedicated time was an important structural change in an otherwise busy schedule. In an early reflection, a 2011 fellow lamented that many medical educators experience a lack of infrastructure to enable them ‘to effectively pursue scholarship as teaching,’ which she described as both time and academic support. She explained:

*Many of us feel that the biggest obstacles [for pursuing educational scholarship] are time and that we don’t have adequate support to do the busy work that is required of us day to day. It is hard to turn anything that we do into scholarship if we don’t have time or help to pursue it.*

This same finding was echoed by a 2012 fellow who explained that finding time to pursue educational scholarship was particularly difficult for clinicians, because they are hired to primarily generate revenue. He indicated that even if clinicians were interested in conducting educational scholarship, the reality is that without dedicated time, educational research would just not get any attention. He explained his reasoning in this way:

*As clinicians, we straddle this world of being interested in teaching, research, and [becoming] better teachers, and also wanting to be a part of the academic environment. [However], the reality is that we are all under the gun to produce, bill and generate revenue.*

Dedicated time allowed participants to reflect upon and analyze their work in new ways, which would not have been possible without the fellowship. The benefit participants received was summed up in an interview by a 2012 participant:

*Having that space reserved for me to stop and think and not be in such a hurry, but reflect on things and slowdown was incredibly valuable. There are fewer and fewer spaces where we can do that stuff. It is worth fighting for.*

Upon graduation, most of the participants indicated that they would continue to provide themselves with protective time to continue their research. For example, a 2014 fellow explained she will continue to carve out this time because it is been fruitful to her career development. Her department chair initially encouraged her to join the fellowship with this way:

*Once you have this skill set, you will have a job for the rest of your life.*

After participating in the fellowship, she sees the value of having been formally trained in educational research and is grateful for the time she was given to develop this skill set.

## Opportunities to engage with others

All of the fellows attributed their success to the interactions they had with other people, although participants differed in who they felt was most influential. Some fellows felt the opportunity to develop a community of like-minded individuals was much needed and helped her succeed. For example, as a 2012 fellow explained, educational research is often undervalued in the health sciences where clinical research is often viewed as the only ‘real’ research. She found it difficult to convince her colleagues of the merit in educational research and benefit to pursuing educational research to academic medicine. In contrast, the fellowship created a community that was refreshing and supportive of her interests. In her interview, she explained that the main benefit of working with others who had similar interests is the feeling of support and the ability to exchange ideas:

*The exchange of ideas was really useful. What most people say about educational research is that it is not clinical research [and in these conversations], their eyes glaze over. It is just not valued. Being with a bunch of people who value [educational research] was great.*

For other fellows, it was the mentoring provided by the Fellowship Director who provided ample opportunities for feedback and assistance. For example, a fellow from 2012 indicated he had plenty of ideas for interesting research projects, but really struggled with ‘follow-through and implementation.’ Being able to talk out loud and get feedback from an expert helped him clarify his own thoughts, and move forward his ideas in ways he couldn’t do alone. In his interview, he explained:

*This fellowship really clicked for me. I work really well when I’m with a group of people who will let me talk for a few minutes, listen to my ideas and then say, “In three minutes, you generated 50 ideas, [in which] 47 of them are garbage. Three of them are probably ok, [but] one of them has some merit. Let’s blow the dust off it and work on that.”*

And finally, a few fellows cited the tutelage of former fellows who remained a part of the fellowship experience as contributory. One participant commented that designing a research question was very difficult for her because it is so unlike conducting research in bench science. Previously, a new drug would be handed to her and she was expected to run tests on it without knowing what bigger questions or assumptions were being tested. She recognized that in educational research formulating the research question is an important step to the research process and one that she struggled with given her bench science research background. Having the former fellows come and talk about their experience helped gauge her own progress in learning to conduct educational research. She indicated that it was ‘*good to hear about previous projects, how they used their time, and the challenges they faced,*’ because she felt that others had already treaded the rocky path.

## Identifying the differences in educational vs clinical research

Participants in this fellowship also developed a deeper understanding of the differences between clinical and educational research and the conceptual tools specific to educational research. Although the curriculum did not specifically address this difference, fellows noted these differences throughout the fellowship experience. According to their initial surveys, most of the participants’ previous experience was in bench science research, which may be why they struggled with certain aspects of transitioning to educational research. For example, a 2012 participant explained *“I had some educational research experience, but still felt very incompetent,”* explaining that she had previously tried to conduct research on her own, but was not successful. This participant, like all but one of the others, began the fellowship programme using bench science research as a framework for thinking and designing their studies. The level of difficulty that some fellows experienced in breaking out of the framework is evidenced in a reflection by a 2011 fellow where the fellow is trying to apply her training from bench science research to make sense of educational research:

*Does educational research still follow the scientific method? How do we deal with all the variables (differences between students) in analyzing our results? Can we draw accurate conclusions with all these variables? How definitive can we be to say that what we did was the reason that we got a specific result?*

Although it was initially difficult for most of the participants to understand the differences between clinical and educational research, what was most challenging was understanding and using theoretical frameworks. Several participants indicated that they had never thought about conceptual frameworks before coming to the fellowship, but later learned that they are typically *‘the basis for the entire [educational] paper/study.’ *Theoretical frameworks seemed like an entirely new concept to the fellows and marked a clear distinction between the two kinds of research. A 2011 fellow expressed her confusion around conceptual frameworks in one of her reflections:

*I’m still not sure I completely understand. Are the conceptual frameworks mentioned in the article [we read] the only ones? Or were those just examples?*

For some fellows, the uncomfortableness they felt with conceptual frameworks never seemed to dissipate. A 2010 fellow anticipated that they would be one of the greatest challenges in moving forward with her scholarship as she began to conduct research on her own, outside the fellowship. This same sentiment was later echoed by a 2011 fellow:

*One of the problems that I struggled with … was that with so many types of conceptual frameworks out there (good and bad), that one really needed to dig into the literature to figure out if it’s a good framework. That seems time consuming to become almost an expert in conceptual frameworks just to be able to apply a few to how you look at your problem. I’m not saying that it isn’t a good idea, but it is difficult as a novice educator.*

Clinical educators and physicians may not be alone in this regard. It is common even for novice educational researchers outside the health sciences to struggle with making sense of using frameworks to analyze and understand their studies. Although the fellows struggled with conceptual frameworks, their level of knowledge around these concepts was enough for the fellows to be successful in producing educational scholarship. Although they still considered themselves novice researchers by the end of the programme, the curriculum seemed to have provided enough information to help them transition.

## Discussion

Our retrospective view of MCG’s Educational Innovation Institute fellowship indicates that it was successful in developing physicians and clinical educators to become educational researchers. In the five years since its inception, the 11 participants who engaged in our programme produced a total of 90 pieces of educationally related scholarship in which all of their training on conducting educationally related research was achieved through the fellowship. Previous criticisms of fellowships, such as the fellowship at MCG, include faculty not producing educational scholarship after completing their programme [11]. And yet, our programme seems to have been successful in this regard.

At the same time, our analysis reinforces the notion that the path to educational researcher is not well understood, and our study raises an important issue. Two of the three mechanisms for fellows’ success point to structural changes in their daily activities. Dedicated time and opportunities to interact with others helped the fellows produce educational scholarship by allowing them to reflect on their ideas and receive feedback. Yet, the curriculum was also an important component. Although the fellowship curriculum did not specifically target the differences between types of research, the fellows made note of these nuanced differences. This finding is important because it provides a clue for how programmes may want to design their curricula to facilitate this transition.

Any curricula to train educational researchers may want to consider explicitly pointing out some of the fundamental differences between clinical and educational research to help fellows navigate this cultural shift, specifically addressing conceptual frameworks. Fundamental differences in clinical and educational research represent the two different cultural approaches to research. How fellows negotiate this difference is an important area in need of further research.

As we move forward in our own programme, we plan to collect more detailed data that will develop a clearer picture of how these differences are negotiated and understood by fellows over time. We plan to revise our evaluation to capture the cognitive changes fellows experience through more qualitative inquiry. We hope to identify the sequence of skills and knowledge developed in educational researchers with a focus on using and understanding conceptual frameworks through qualitative data sources (i. e. additional interviews and focus groups).

## Conclusion

As long as the Accreditation Council for Graduate Medical Education and the Liaison Committee on Medical Education requires medical faculty to engage in scholarly activity for accreditation and promotion, fellowships such as the educational research fellowship track may be one of the most viable vehicles for producing educational scholars. However, like any process, the transition from clinical educator or physician to educational researcher needs to be studied more systematically. The results of this study provide a useful first step in identifying the mechanisms that may produce educational researchers. We now need to focus our attention more deliberately on capturing the incremental shifts fellows achieve and the developmental process our fellows experience.

## References

[1] Thompson B, Searle N, Gruppen L (2011). A national survey of medical educaiton fellowships. Med Educ Online.

[2] Grady E, Roise A, Barr D (2012). Defining scholarly activity in graduate medical education. J Grad Med Educ.

[3] McLean M, Cilliers F, van Wyk J (2008). Faculty development: yesterday, today and tomorrow. Med Teach.

[4] Steinert Y, Nasmith L, McLeod P (2003). A teaching scholars program to develop leaders in medical education. Acad Med.

[5] Gruppen L, Frohna P, Anderson R (2003). Faculty development for educational leadership and scholarship. Acad Med.

[6] Searle NS, Hatem C, Perkowski L (2006). Why invest in an educational fellowship program. Acad Med.

[7] Hitchcock M, Stritter F, Bland C (1992). Faculty development in the health professions: conclusions and recommendations. Med Teach.

[8] Richardson D, Villarosa M, Palladino C (2011). MedEdPORTAL module guides evaluation of faculty fellowship.

[9] Gruppen L, Simpson D, Searle N (2006). Educational fellowship programs: common themes and overarching issues. Acad Med.

[10] Glaser BG, Strauss AL (2009). The discovery of grounded theory: strategies for qualitative research.

[11] Steinert Y (2011). Faculty development: The road less traveled. Acad Med.

